# 

**Published:** 1909-04-17

**Authors:** 


### BOOKS RECEIVED.

Spottiswoode and Co.

"The Medical Register, 1909."
"The Dentists' Register, 1909."

J. Nisbet and Co.

"The Position of Abdominal Hysterectomy in London." By J. Bland-Sutton, F.R.C.S.

J. B. Lippincott Co.

"Diseases of the Nose, Throat, and Ear." By Francis R. Packard, M.D.

"Diseases of the Digestive Canal." By Dr. Paul Cohnheim. Edited and translated by Dudley Fulton, M.D.

"Gynæcological Diagnosis." By George Winter. Edited by John G. Clark, M.D.

Longmans, Green and Co.

"Heredity and Disease, a Discussion." Edited by J. Nachbar, M.D.

### THE GRADUATES' MEDICAL SCHOOLS.

DIARY FOR THE WEEK APRIL 19 TO APRIL 24.

**MEDICAL GRADUATES' COLLEGE AND POLYCLINIC, 22 Chenies Street, W.C.**

**At 4 p.m.**

**April 19,** Dr. George Pernet. **Skin.**
**April 20,** Dr. Newton Pitt, **Medical.**
**April 21,** Mr. James Berry, **Surgical.**
**April 22,** Sir John Hutchinson, **Surgical.**
**April 23,** Mr. Gay French, **Ear, Nose, and Throat.**

### ANSWERS TO CORRESPONDENTS.

Dr. W. J. Lawrie writes: In reading over some prescriptions in a German "Rezeptformular" I came across an abbreviation I do not quite understand. One formula was—

R. Acid. acetyl-salicyl. 0,5 (-1,0)
D. tal. dos. No. x.
S. 3-5 mal täglich ein Pulver zu nehmen (Für Erwachsene).

Does the "D." mean "divisa," or "dirige," or what? Another formula has "M.D. in vitro nigr." What does "M.D." exactly mean?

[In German prescriptions "D." means "Dona"; "M.D." signifies "Misce Dona"; "M.D.S." corresponds to the English "M. ft. mist.," and signifies "Misce dona signa."]

THE HOSPITAL April 17, 1909.

*Name* ..............................

*Address* ..............................

..............................

This Coupon must accompany manuscript or contributions intended for The Hospital.

